# Supplementary material for: Coral tissue depth reconstructed using skeletal microstructural offsets is driven by environmental stress
Source: Commun Earth Environ. 2026 Jan 19;7(1):136. doi: 10.1038/s43247-025-03114-2 (PMC12880916; doi:10.1038/s43247-025-03114-2)
Supplement: Supplementary file 2 — Supplementary Information [file 43247_2025_3114_MOESM2_ESM.pdf]

Coral tissue depth reconstructed using skeletal microstructural offsets is driven by environmental stress

James Vincent*, Tom Sheldrake*

james.vincent@unige.ch
thomas.sheldrake@unige.ch

Department of Earth Sciences, University of Geneva, Genève, Switzerland

Supplementary Information

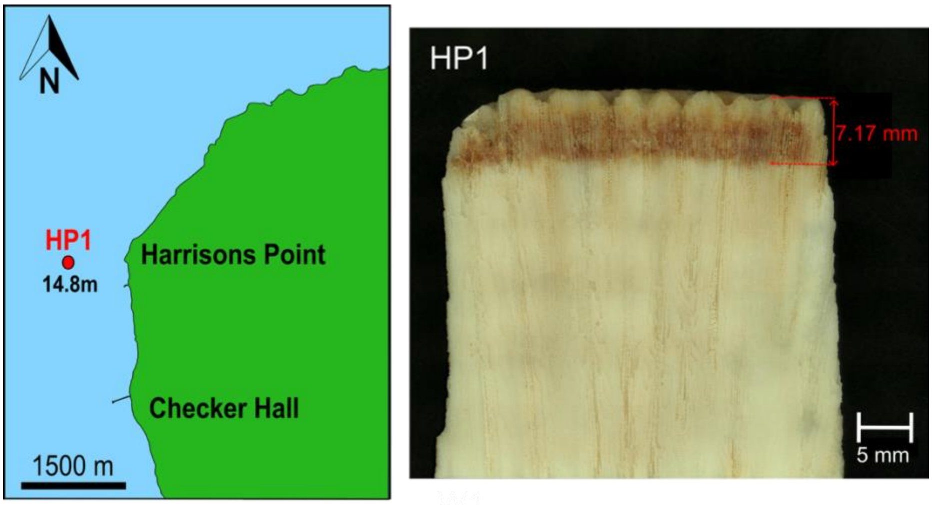

S.1. Map of the North-West coast of Barbados with HP1 sample location and image of the tissue staining. The red circle in the left panel indicates the sample location and corresponding water depth. The tissue staining of HP1 is illustrated in the right panel by the red arrows. Map and sample image have been adapted from Vincent & Sheldrake, 2025[1].

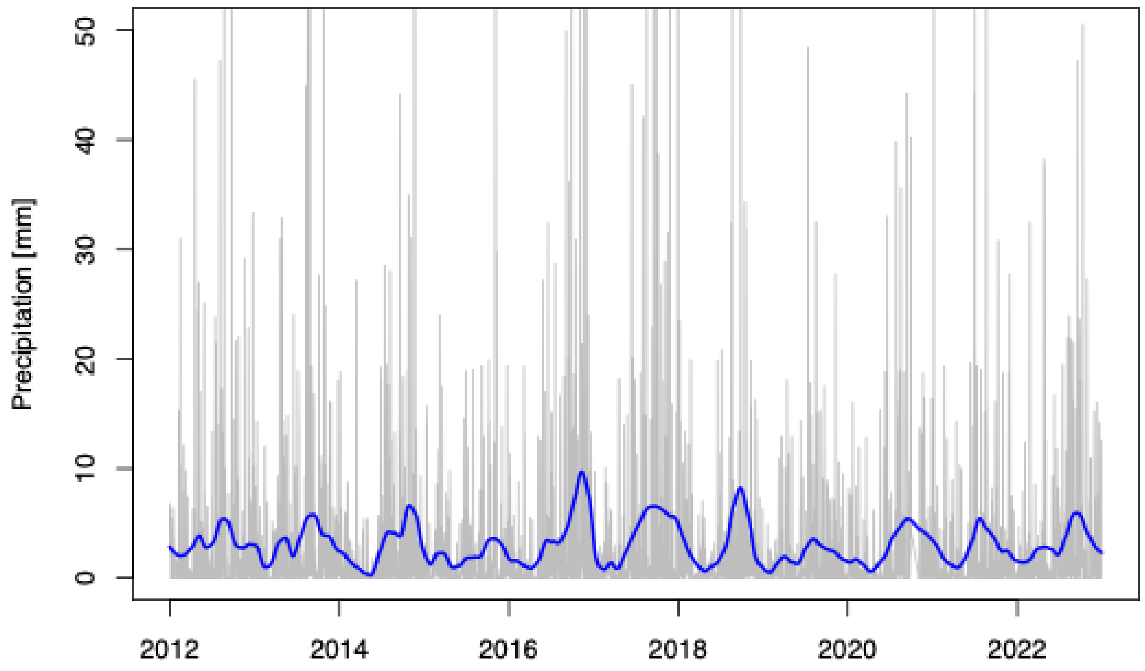

S.2. Raw and smoothed daily precipitation from Grantley Adams Airport, Barbados, from January 2012 to July 2022. Daily precipitation (grey) and LOESS-smoothed precipitation (blue) are plotted over time. The smoothing span of 182 days (6 months) was selected to visualise seasonal to interannual variability in precipitation. The plot shows 11 seasonal cycles corresponding to 11 annual rainy seasons in the Caribbean.

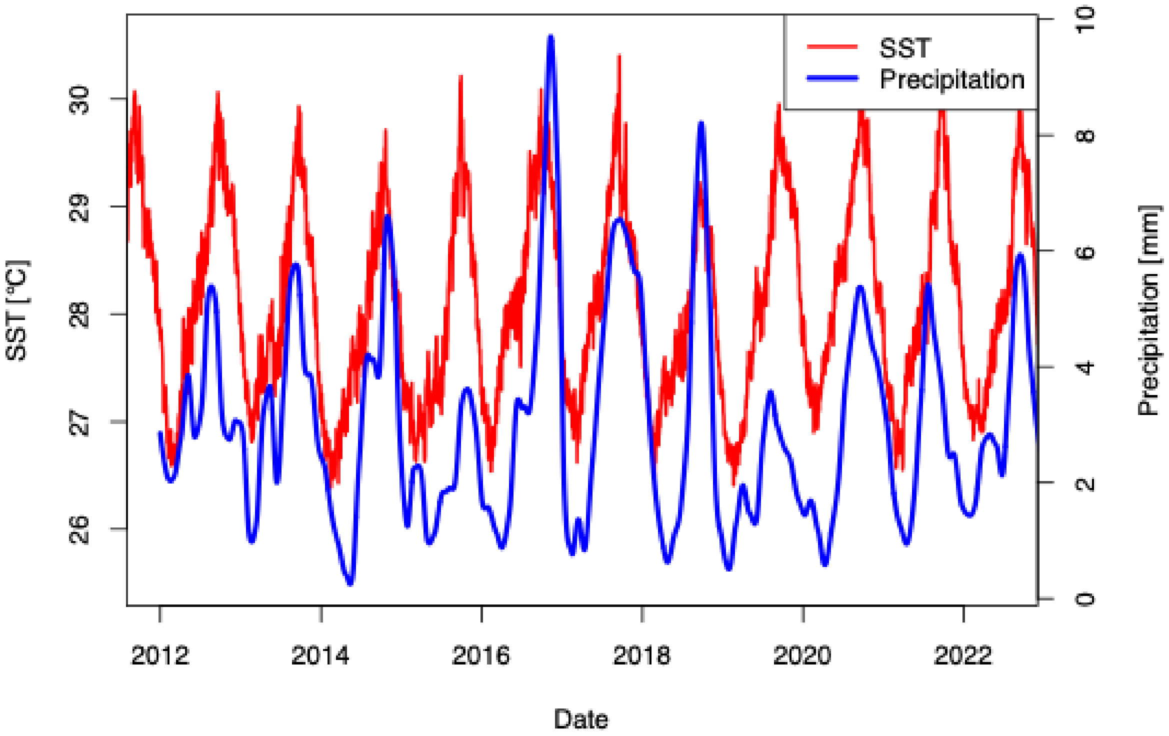

S.3. Sea surface temperature (SST) and smoothed precipitation from January 2012 to July 2022. Daily SST (red) and LOESS-smoothed precipitation (blue) are plotted over time. SST is shown on the left y-axis and precipitation on the right y-axis. The smoothing span of 182 days (6-months) was selected to highlight seasonal to interannual variability in precipitation and SST. This comparison highlights the seasonal and interannual co-variation between SST and precipitation in Barbados covering the timeseries reconstructed from our sample (HP1). Note that the peaks and troughs of the precipitation and SST data are slightly out of sync.

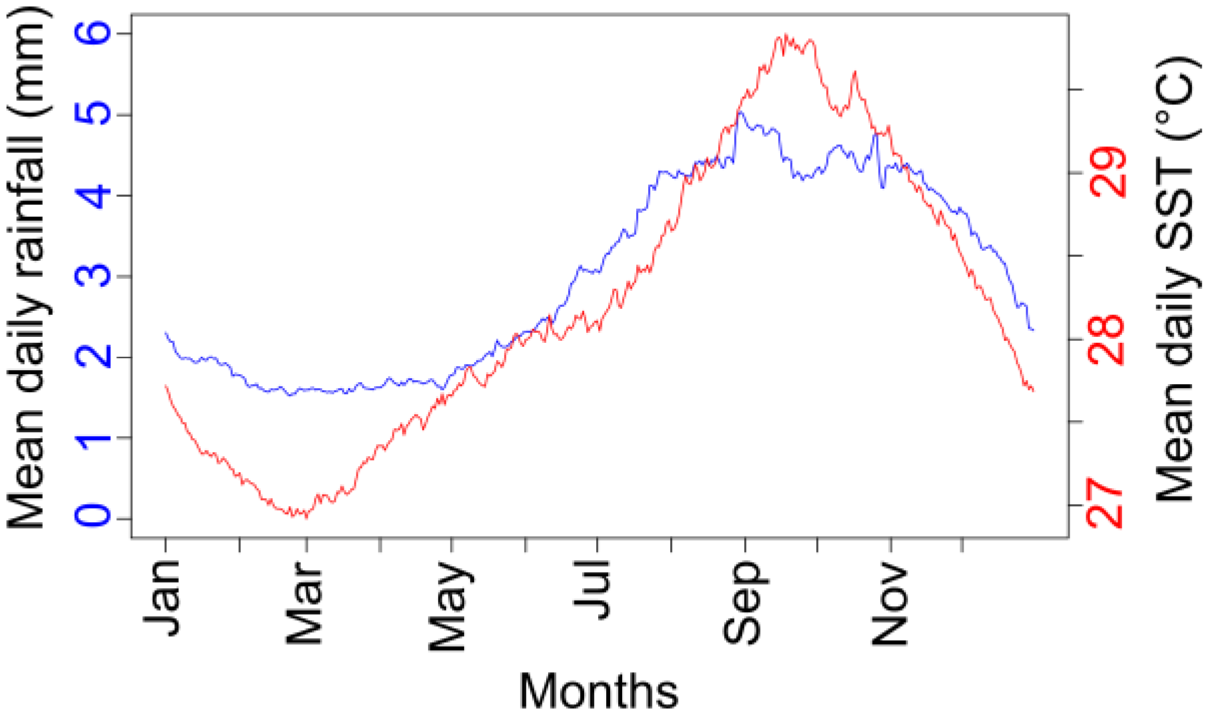

S.4. Mean daily SST and precipitation from 2012 to 2022. SST and precipitation are represented by the red and blue lines, respectively. It was necessary to smooth the precipitation data by a running mean window of 60 days to present a seasonal signal.

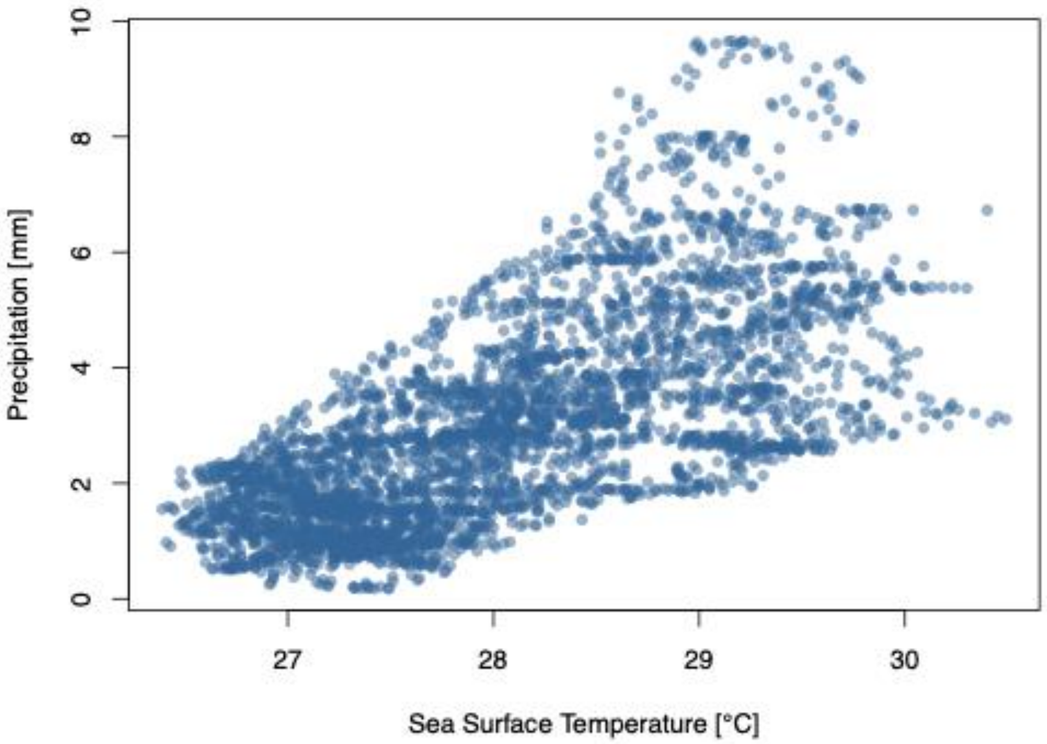

S.5. Scatterplot showing the relationship between sea surface temperature (SST) and smoothed precipitation. Each point represents a daily value of mean SST and the corresponding LOESS-predicted precipitation between January 2012 to July 2022. Point transparency indicates overlapping observations. The Pearson correlation coefficient ($r = 0.69$) suggests a moderate positive relationship between SST and precipitation.

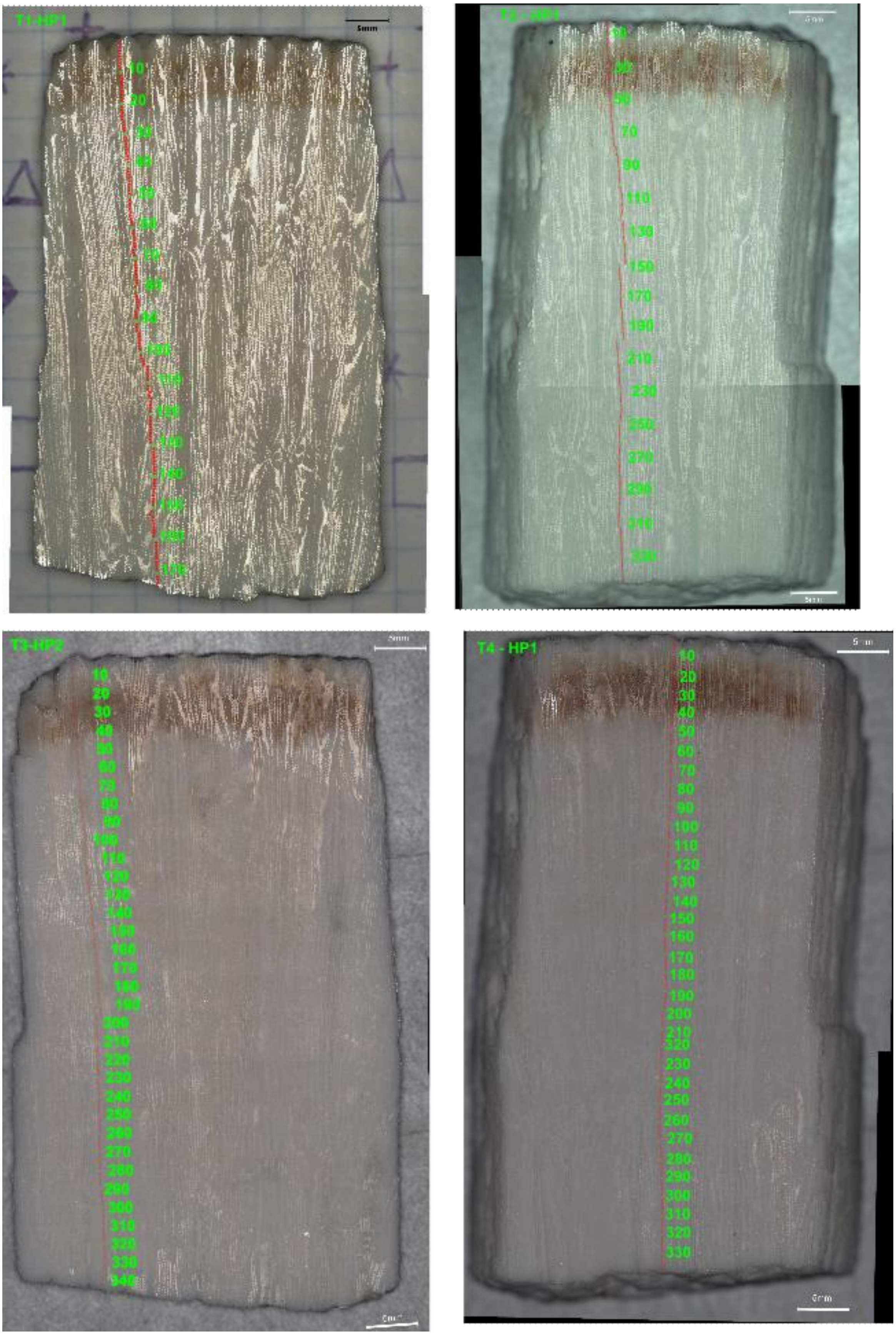

S.6. Four LA-ICP-MS transects from four analytical sessions on HP1. The top left panel corresponds to transect 1 (T1) which contains 173 points at 360-micron spatial resolution. The top right panel corresponds to transect 2 (T2) which contains 345 points at 180-micron spatial resolution. Transects 3 and 4 (T3 and T4) are shown in the bottom left and right panels and contains 343 and 399 spots, respectively, at 180-micron spatial resolution. Note that the sample surface was repolished between analytical sessions (see methods) to reveal a continuous theca structure.

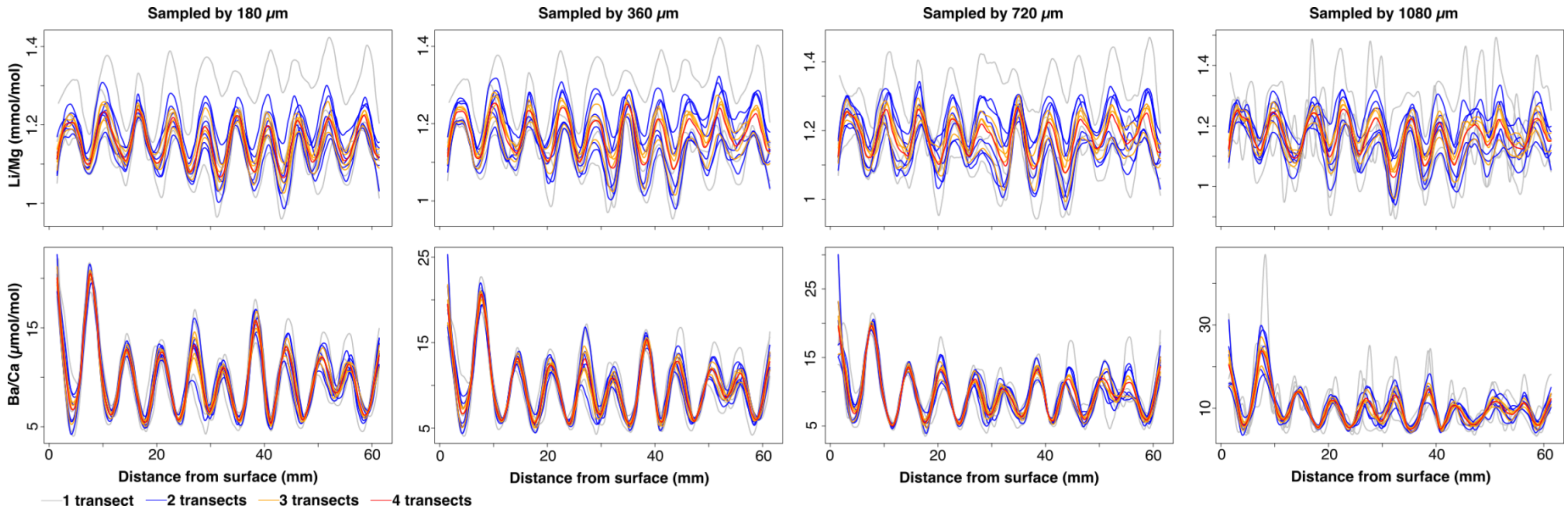

S.7. LOESS smoothed transects (T1 - T4) shown at different sampling intervals and with different combinations of transects.

For both the Li/Mg (top row) and Ba/Ca (bottom row) profiles, the results show that the averaged signal converges toward the true average signal (red line) of the sample with an increasing number of transacts, even when spatial resolutions is reduced. The higher special resolution smooths out natural variability in the sample.

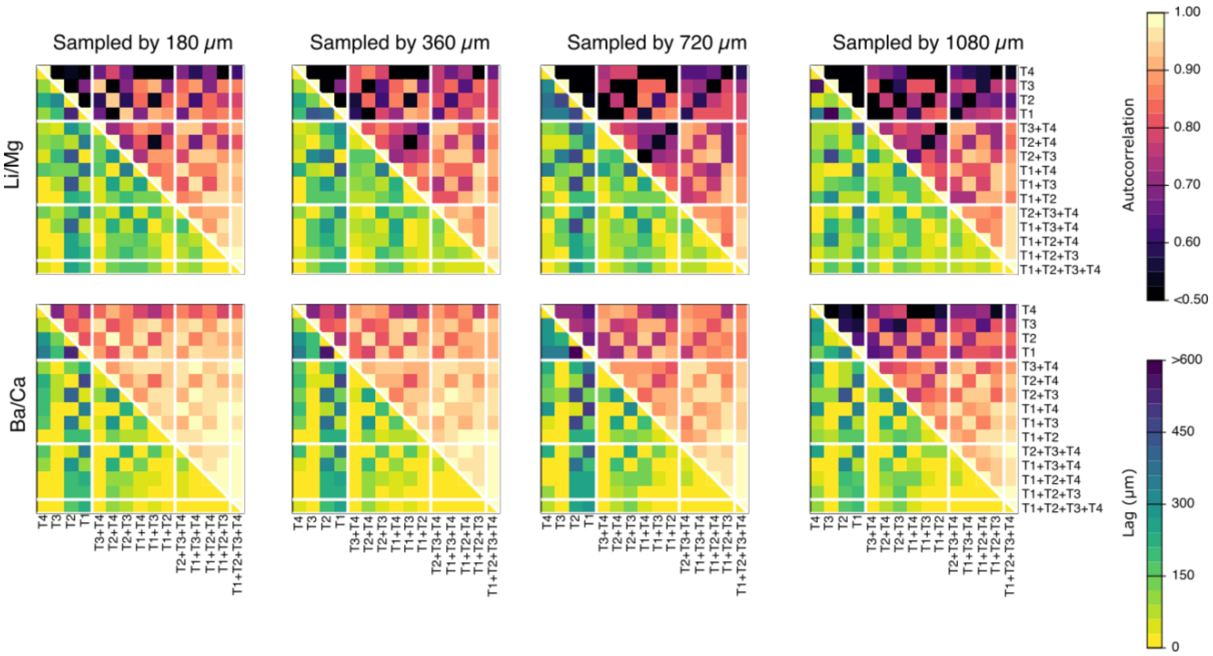

S.8. Cross-correlation matrices of Li/Mg (top row) and Ba/Ca (bottom row) between LOESS smoothed analytical transects (T1 - T4) at different (sampled) spatial resolutions and transect combinations. T1 was analysed at a spatial resolution of 360-microns whilst T2, T3, and T4 were analysed at 180-micron spatial resolutions.

Results from the correlation matrix show that individual transects are not well correlated even at higher spatial resolutions in T2, T3, and T4, with large lags (bottom left triangles) and low autocorrelation (upper right triangles). As the number of transects increases, both the lag decreases and autocorrelation increases signifying a convergence to the mean value for the sample. When combining three transects there is very little lag between signals, especially for Ba/Ca, and below 150 microns for Li/Mg.

Comparing the different combination of three transects to combining all four transects shows perfect autocorrelation for Ba/Ca, and excellent autocorrelation for Li/Mg (>0.9). This is evident even when the transects are down-sampled to a resolution of 720 microns. Consequently, we are confident that the combination of T1-T4 successfully reveals the true average signal of the sample.

Isotope	Mean [µmol/mol]	2SD [µmol/mol]	2RSD [%]	Accuracy [%]
Li7	9.4	1.1	11.2	115.8
Mg25	4245.2	352.3	8.3	107.8
Ba137	8.5	0.7	8.5	113.4

ST.1.1. The analytical accuracy and precision of the JCp-1-NP reference material. All calculations use the total combined JCp-1-NP measurements (n = 36). The precision is calculated as two times the relative standard deviation from the mean (2RSD) and is expressed as a percentage. The accuracy is calculated using the official reference material values[2].

Isotope	Mean [µmol/mol]	2SD [µmol/mol]	2RSD [%]	Accuracy [%]
Li7	9.5	1.3	13.9	118.1
Mg25	4300	350.7	8.7	102.8
Ba137	8.8	0.4	4.3	112.3

ST.1.2. The analytical accuracy and precision of the JCp-1-NP reference material. All calculations use the JCp-1-NP measurements from analytical session 28-10-2022 (n = 14). The precision is calculated by the two times the relative standard deviation from the mean (2RSD) which was taken from Flöter et al (in review)[3] (n = 226, 250, 225 for Li/Ca, Mg/Ca and Ba/Ca respectively) and is expressed as a percentage. The accuracy is calculated using the official reference material values[2].

Isotope	Mean [µmol/mol]	2SD [µmol/mol]	2RSD [%]	Accuracy [%]
Li7	9.5	0.8	9.1	113
Mg25	4300	304.3	7	110
Ba137	8.8	0.8	9.3	114

ST.1.3. The analytical accuracy and precision of the JCp-1-NP reference material. All calculations use the JCp-1-NP measurements from analytical session 10-06-2025 (n = 9). The precision is calculated by the two times the relative standard deviation from the mean (2RSD) which was taken from Flöter et al (in review)[3] (n = 226, 250, 225 for Li/Ca, Mg/Ca and Ba/Ca respectively) and is expressed as a percentage. The accuracy is calculated using the official reference material values[2].

Isotope	Mean [µmol/mol]	2SD [µmol/mol]	2RSD [%]	Accuracy [%]
**Li7**	9.5	0.7	7.3	115.9
**Mg25**	4300	148.8	3.5	109.4
**Ba137**	8.8	0.8	10.1	112

ST.1.4. The analytical accuracy and precision of the JCp-1-NP reference material. All calculations use the JCp-1-NP measurements from analytical session 04-08-2025 (n = 8). The precision is calculated by the two times the relative standard deviation from the mean (2RSD) which was taken from Flöter et al (in review)[3] (n = 226, 250, 225 for Li/Ca, Mg/Ca and Ba/Ca respectively) and is expressed as a percentage. The accuracy is calculated using the official reference material values[2].

Isotope	Mean [µmol/mol]	2SD [µmol/mol]	2RSD [%]	Accuracy [%]
**Li7**	9.5	0.8	8.9	114.4
**Mg25**	4300	194	4.5	110
**Ba137**	8.8	1	11.3	115.7

ST.1.5. The analytical accuracy and precision of the JCp-1-NP reference material. All calculation uses the JCp-1-NP measurements from analytical session 06-08-2025 (n = 7). The precision is calculated by the two times the relative standard deviation from the mean (2RSD) which was taken from Flöter et al (in review)[3] (n = 226, 250, 225 for Li/Ca, Mg/Ca and Ba/Ca respectively) and is expressed as a percentage. The accuracy is calculated using the official reference material values[2].

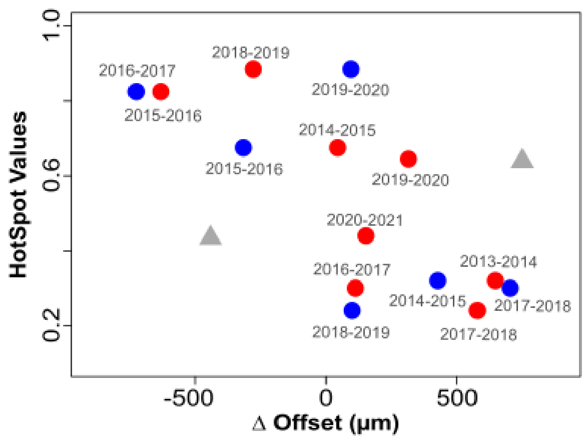

S.9: Scatter plot showing the relationship between the Δ offset and HotSpot values. Red and blue circles correspond to the Δ offset values between consecutive years (labelled in grey) for warm/wet-seasons and dry/cold-seasons, respectively. Cold/dry-season Δ offsets are plotted against the HotSpot values of the intervening warm/wet-season. Conversely, the warm/wet-season Δ offsets are plotted against the HotSpot values of the latter year. Grey triangles represent years in which the Δ offset included anomalous measurements from 2021 which are influenced by the volcanic eruption in the dry/cold-season of 2021.

Supplementary References

1. Vincent, J. & Sheldrake, T. Micro-CT analysis reveals porosity driven growth banding in Caribbean coral Siderastrea siderea. *Sci Rep* **15**, 6063 (2025).
2. National Institute of Advanced Industrial Science and Technology (AIST), G. S. of J. *Certified Geochemical Reference Material: GSJ CRM JCp-1 Coral (Porites Sp.).* https://gbank.gsj.jp/geostandards/Certificate/PDF/eJCp1.pdf (2019).
3. Flöter, S. *et al.* A long-term study of the reference material JCp-1-NP: new and compiled LA-ICP-MS elemental compositional data. *Scientific Data[in review]* (2025).
